# Comparison of early vs. routine removal of abdominal drainage tube after laparoscopic appendectomy for perforated appendicitis: a retrospective cohort study

**DOI:** 10.3389/fsurg.2025.1617312

**Published:** 2025-07-18

**Authors:** Jun He, Gang Qian, Yefei Mao, Lei Gao

**Affiliations:** Department of General Surgery, Zhangjiagang Third People’s Hospital, Zhangjiagang, China

**Keywords:** abdominal drainage tube, laparoscopic appendectomy, perforated appendicitis, postoperative complication, intraabdominal abscess

## Abstract

**Background:**

The utility of abdominal drainage is common in laparoscopic appendectomy (LA) for acute appendicitis with perforation to prevent postoperative complications, such as intraabdominal abscess (IAA) and stump fistula. Nevertheless, the drain tube placement is considered to be associated with postoperative IAA formation in cases of complicated appendicitis. Our study aims to determine whether early removal of abdominal drainage tube after LA can improve prognosis for patients with perforated appendicitis.

**Methods:**

A total of 182 patients who underwent abdominal drainage tube placement during LA due to acute appendicitis with perforation were divided into experimental group and control group by random number table method, including 87 patients in the experimental group and 95 patients in the control group. Patients in the experimental group had their abdominal drainage tube removed within 48 h after surgery. Patients in the control group removed the drainage tube after 48 h routinely. Variables of demographic and clinical characteristics of these patients between the two groups were analyzed. Postoperative outcomes, including overall complications, IAA, superficial surgical site infection (SSI), stump fistula, ileus, bleeding, postoperative length of stay (LOS), hospitalization costs and readmission to hospital, were compared.

**Results:**

These two groups were similar regarding demographic and perioperative clinical characteristics like age, sex, duration of symptoms and hematological examination indicators (*P* > 0.05). Although there was no significant difference in superficial SSI and ileus between the two groups (*P* > 0.05), patients in the experimental group was associated with a lower rate of overall complications (3.4% vs. 17.9%, *P* = 0.002), declined incidence of IAA (3.4% vs. 11.6%, *P* = 0.040), a shorter LOS [4 (4,4) vs. 6 (5,6) days, *P* < 0.001] and less hospitalization costs [9,705 (8,621−10,402) vs. 10,851 (9,704−11,752) CNY, *P* < 0.001] compared with patients in the control group. No stump fistula and intraabdominal bleeding occurred in both groups. There was no significant difference in readmission rate within 30 days after surgery between the two groups (*P* = 0.684).

**Conclusions:**

It is safe and effective to remove abdominal drainage tube within 48 h after LA for patients with perforated appendicitis. This approach can accelerate the recovery time, decline the incidence of IAA and reduce hospitalization costs.

## Introduction

Acute appendicitis is the most common abdominal surgical emergency in the world (1). Most studies have divided acute appendicitis into uncomplicated and complicated appendicitis based on the severity of inflammation (2, 3). Perforated appendicitis belongs to complicated appendicitis. Laparoscopic appendectomy (LA) is the preferred treatment for both uncomplicated and complicated appendicitis with a shorter length of stay (LOS) and a lower incidence of postoperative complications (4–7).

Complicated appendicitis is considered to be a risk factor for postoperative complications, prolonged LOS and readmissions (8). For decades, the abdominal drainage placement is thought to be an effective approach to prevent the accumulation of inflammatory exudate, resulting in a declined incidence of intraabdominal abscess (IAA). However, both the 2020 WSES guideline and the 2024 SAGES guideline recommend no use of drains following appendectomy for complicated appendicitis in adult patients and children, with a low quality of evidence (5, 7). Recent studies have reported similar results that abdominal drainage fails to prevent overall complications and specific complications such as IAA formation, and the placement of drainage tube is associated with prolonged LOS (9–11).

There are around 33% to 44% patients with complicated appendicitis reported to receive abdominal drainage during surgery in previous retrospective studies (10, 12). Due to the severe inflammation of perforated appendicitis, the drain placement is more frequently utilized to monitor postoperative abdominal bleeding, appendix stump fistula and prevent abscess formation. For those patients who undergo abdominal drainage management, they are at a higher risk of postoperative complications and longer LOS. The aim of this study is to determine whether early removal of abdominal drainage tube within 48 h after surgery can improve prognosis for those patients.

## Methods

### Patients

Inclusion criteria were as follows: age over 18 years; American society of Anesthesiologists (ASA) score I and II; diagnosis of complicated appendicitis with perforation; abdominal drainage tube placement during LA.

Exclusion criteria were as follows: perforated appendicitis with abscess formation or diffuse peritonitis; moderate or severe immunocompromised patients; Intensive Care Unit stay; history of abdominal surgery; conversion to open appendectomy.

With the approval of the Ethics Committee of the Zhangjiagang Third People's Hospital (ZJGSY-2022-13), a total of 182 patients who met the inclusion criteria were included in this study between April 2022 and March 2025. All patients provided written informed consents. The diagnosis of perforated appendicitis was based on medical history, clinical symptoms, computed tomography (CT) images and observation during laparoscopy. Abscess formation or diffuse peritonitis was diagnosed by clinical symptoms, preoperative CT images and laparoscopic findings. The diagnosis and severity of immunocompromised patients referred to Coccolini's guidelines (13).

### Perioperative management

All patients underwent similar preoperative preparation and anesthesia methods. All surgeries were conducted by surgeons with experience of at least 50 LA cases. Intracorporeal suture was used to close stump. After disconnecting the appendiceal at the root, the cecal wall around the appendiceal base was intermittently sutured under laparoscopy, and then the surrounding tissue was used for suturing and embedding. Suction alone without abdominal lavage was utilized to prevent the bacteria growth with the existence of peritoneal effusion. Abdominal drainage tube was then placed during surgery and surgeons could estimate whether there was bleeding or fistula based on the characteristics of fluid in the drainage tube postoperatively. Early mobilization and liquid intake (2 h after surgery) were encouraged. Patients were advanced to a soft diet 6 h later and a normal diet within 48 h if possible. All patients were randomly divided into an experiment group and a control group using a random number table method. Patients in the experiment group removed abdominal drainage tube within 48 h after LA if intraabdominal bleeding or fistula were not detected according to the characteristics of fluid in the drain tube. Ultrasound examination would be performed to determine whether there was fluid accumulation around the appendix stump if necessary. Whereas, the criterion for routine drain removal in the control group was serosanguinous fluid <40 ml/day for at least two consecutive days. All patients received a two-day intravenous antibiotic treatment after LA, and then switched to oral antibiotics. Intravenous antibiotic treatment would continue to be adopted if the IAA was diagnosed. The main antibiotic used in our center was ceftriaxone. If patients were allergic to cephalosporin drugs, levofloxacin was chosen. Patients were discharged home if they had no complications.

### Outcomes

The demographics and perioperative clinical characteristics of the patients are collected, including age, sex, duration of symptoms, preoperative fever with body temperature over 38.5°C, white blood cell count, neutrophil percentage, C-reactive protein and appendiceal diameter.

The primary outcomes measured were postoperative complications, including IAA, superficial surgical site infection (SSI), stump fistula, intraabdominal bleeding and ileus, within 30 days after surgery. IAA, stump fistula and ileus were diagnosed by clinical symptoms and ultrasound or CT findings. Superficial SSI was defined as clinical pus formation or erythematous change of the wound.

Secondary outcomes included postoperative LOS, hospitalization costs and readmission to hospital within 30 days after surgery.

### Statistical analysis

Statistical analysis was performed via SPSS statistical software (version 25.0, IBM Corporation, New York, USA). The measured data are expressed as the mean (SD) or median (IQR) and were analyzed by the *t*-test or the Mann Whitney test. The enumeration data were analyzed by the *χ*^2^ test. A *P*-value <0.05 indicated a statistically significant difference.

## Results

A total of 182 patients who underwent abdominal drainage tube during LA for perforated appendicitis were eligible for this study. Eighty-seven patients were included in the experimental group, and 95 patients were included in the control group.

The baseline demographics and characteristics of the two groups are shown in Table 1. In the experimental group, 58.6% (51 in 87) of the patients were male and the mean (SD) age was 46.94 (14.84) years. The median (IQR) duration of symptoms was 18 (9–31) h. Eight patients had experienced a fever with body temperature over 38.5°C at their admissions. The mean (SD) preoperative white blood cell count was 13.03 (3.76) *10^9^/L and the mean (SD) C-reactive protein was 57.8 (24.5) mg/L. The median (IQR) neutrophil percentage was 85.5 (79.6–89.6) % and the median (IQR) appendiceal diameter measured in CT images was 1.0 (1.0–1.5) cm. In the control group, 52.6% (50 in 95) of the patients were male and the mean (SD) age was 50.44 (14.29) years. The median (IQR) duration of symptoms was 24 (8–34) h. Thirteen patients had experienced a fever with body temperature over 38.5°C at their admissions. The mean (SD) preoperative white blood cell count was 12.67 (3.95) *10^9^/L and the mean (SD) C-reactive protein was 64.0 (32.0) mg/L. The median (IQR) neutrophil percentage was 86.7 (80.9–90.7) % and the median (IQR) appendiceal diameter measured in CT images was 1.4 (1.0–1.5) cm. No significant differences were found in age (*P* = 0.417), sex (*P* = 0.107), duration of symptoms (*P* = 0.475), preoperative fever (*P* = 0.344), white blood cell count (*P* = 0.536), neutrophil percentage (*P* = 0.111), C-reactive protein (*P* = 0.148) and appendiceal diameter (*P* = 0.135) between the two groups.

**Table 1:** Baseline demographics and characteristics of patients undergoing drainage tube placement during laparoscopic appendectomy for perforated appendicitis.

Group	Experimental group (*n* = 87)	Control group (*n* = 95)	*P* value
Sex (male/female)	51/36	50/45	0.417
Age (y)	46.94 (14.84)	50.44 (14.29)	0.107
Duration of symptoms (h)	18 (9–31)	24 (8–34)	0.475
Fever (yes/no)	8/79	13/82	0.344
White blood cell count (*10^9^/L)	13.03 (3.76)	12.67 (3.95)	0.536
Neutrophil percentage (%)	85.5 (79.6–89.6)	86.7 (80.9–90.7)	0.111
C-reactive protein (mg/L)	57.8 (24.5)	64.0 (32.0)	0.148
Appendiceal diameter (cm)	1.0 (1.0–1.5)	1.4 (1.0–1.5)	0.135

Values are presented as mean (SD), median (IQR) or number.

As shown in Table 2, there were no significant differences in superficial SSI (0 vs. 2, *P* = 0.498), and ileus (0 vs. 4, *P* = 0.122) between the two groups. No case of stump fistula or intraabdominal bleeding was found in both groups. However, the incidence of IAA in the experimental group was lower than that in the control group (3 vs. 11, *P* = 0.040). The incidence of overall complications in the experimental group was much lower than that in the control group (3 vs. 17, *P* = 0.002). The median (IQR) postoperative LOS was 4 (4−4) days in the experimental group and 6 (5–6) days in the control group. The median (IQR) hospitalization costs was 9,705 (8,621–10,402) CNY in the experimental group and 10,851 (9,704–11,752) CNY in the control group. There were significant differences in postoperative LOS (*P* < 0.001)and hospitalization costs (*P* < 0.001) between the groups. Two patients in the experimental group and three patients in the control group were admitted because of IAA. The other one patient in the control group was admitted to hospital due to severe diarrhea within 30 days after surgery. No significant difference was found in readmission rate between the two groups (*P* = 0.684).

**Table 2:** Outcomes of patients between the two groups within 30 days after surgery.

Group	Experimental group (*n* = 87)	Control group (*n* = 95)	*P* value
Overall complications (*n*)	3	17	**0** **.** **002**
Superficial SSI (*n*)	0	2	0.498
IAA (*n*)	3	11	**0** **.** **040**
Appendiceal stump fistula (*n*)	0	0	NA
Intraabdominal bleeding (*n*)	0	0	NA
Ileus (*n*)	0	4	0.122
Postoperative length of stay (d)	4 (4–4)	6 (5–6)	**<0** **.** **001**
Hospitalization costs (CNY, ￥)	9,705 (8,621–10,402)	10,851 (9,704–11,752)	**<0** **.** **001**
Readmission to hospital (*n*)	2	4	0.684

Values are presented as median (IQR) or number.

SSI, surgical site infection; IAA, intraabdominal abscess.

The *P* values <0.05 were highlighted in the bold values.

## Discussion

Recent studies reported an adverse effect of postoperative abdominal drainage after LA, such as higher incidence of complications and prolonged LOS (9–12). However, there were few studies reporting the influence of the timing of drainage tube removal on prognosis after LA. Former studies had demonstrated that early drain removal had shown better outcomes after hepatectomy and pancreatectomy (14, 15). Our study demonstrated that early removal of abdominal drainage tube after LA for perforated appendicitis could decline postoperative LOS, hospitalization costs and the incidence of complications like IAA, with the benefits of monitoring postoperative intraabdominal bleeding and appendix stump fistula directly.

Mulita reported that the incidence of IAA in patients with complicated appendicitis after LA was 5.19% and that after open appendectomy was 7.07% (16). Mao reported 5.4% patients developed post-operative IAA following appendectomy (17). Postoperative abdominal drainage tube was a controversial topic for complicated appendicitis to decrease the incidence of IAA for decades. Pakula reported that the use of drainage in patients with perforated or gangrenous appendicitis during LA had decreased rates of pelvic abscess (6% vs. 20%), comparing to no use of drainage (18). On the contrary, in Human's randomized controlled trial, the postoperative drain made no significant difference in outcomes for complicated appendicitis, and overall complications rate reported in their study was 26% (9 in 34) (19). Liao also reported that the abdominal drainage increased the risk of overall complications and failed to decrease the risk of IAA (9). The similar adverse prognosis of drain placement after appendectomy were both reported in Alabbad's and Fujishiro's studies (11, 20). In the latest update of a Cochrane review, approximately 113 (57–221 participants) out of 1,000 participants in the drainage group developed intraperitoneal abscess, compared with 104 out of 1,000 participants in the no-drainage group (21). Our study showed the similar results with an overall complications rate of 17.9% (17 in 95) and an IAA rate of 11.6% (11 in 95) when the drain tube was placed and removed routinely.

Timing of drainage removal was not mentioned in the previous studies, and there was no worldwide consensus on when to remove the drain tube after LA. In practice, the decision of drainage tube placement and removal was at the discretion of the surgeons. Up to 60.1% patients with perforated appendicitis underwent intraperitoneal drain placement during LA in Alabbad's retrospective study (11). During the period of abdominal drainage tube placement, problems such as erosion, obstruction, drain entrapment or loss due to displacement, kinking or migration, could be existent, leading to the postoperative collections and abscess formation. Early removal of drainage tube was performed after confirming no bleeding or stump fistula in our study, reducing the issues of the routine placement of drainage tube. This may explain the lower incidence of overall complications and IAA formation in patients accepting early removal of drainage tube. To some extent, early removal of drainage tube may have similar outcomes to no drain placement in the literature reported. Compared to no drain placement, early removal of drainage tube had an advantage in that it allowed for intuitive observation of intraabdominal bleeding and fistula, although these complications did not occur in our study. Therefore, if a drainage tube must be placed during LA, early drain removal is recommended after confirming no existence of bleeding or stump leakage.

Early removal of drainage tube was associated with shorter LOS and less hospitalization costs in our study, mainly because patients in our study were discharged from hospital only if there were no complications, and patients who underwent early removal of drainage had a lower incidence of complications, especially the IAA formation. Meanwhile, due to the complexity and inconvenience of drainage tube management, patients and surgeons were more accepting of patients being discharged after drain removal, which also led to prolonged LOS and higher hospitalization costs for patients in our control group. In patients undergoing routine removal of drainage tube, the LOS and costs were both higher, which is similar to the results of previous studies on outcomes of drain vs. no drain. Li reported that abdominal drainage may increase the LOS by 2.17 days compared to no drainage (22). Another meta-analysis reported a longer postoperative LOS and higher overall incidence of postoperative complications as well (23). Voglion also reported that the use of abdominal drains after LA was associated with longer hospitalization in their single-center retrospective study (24). We also found that the readmission rate of patients undergoing early drainage tube removal was similar with those patients undergoing routine drainage management, indicating that it was safe to remove the drainage tube within 48 h after LA for perforated appendicitis.

This study had several limitations. Firstly, this was a single-center retrospective study with a small sample size, which introduced some biases. Randomized controlled trials with large sample sizes are need to be conducted to help eliminate these biases in future. Secondly, the standard for routine removal of drainage tube in our control group is determined based on our single-center's experience, and different surgeons and centers have different extraction standards. For example, the criterion for drain removal was serosanguinous fluid <50 ml/day in Liao's study (9). And in Alabbad's study, drains were removed when the output reached a minimal amount (11). The different management of postoperative drain may result in different outcomes. In the future, it is essential to establish worldwide standards for the removal of drainage tubes after LA to help improve the prognosis. Thirdly, the severity of appendicitis, preoperative and postoperative antibiotic therapy varied among patients. These factors may have varying impacts on prognosis and timing of drainage tube removal. This retrospective study failed to discover the connection between these factors and outcomes after LA. A well-designed randomized control trial could compensate for the inadequacies of this retrospective study. Finally, all patients included in this study were ASA score I and II, and their physical condition was relatively healthy. This meant that the conclusions obtained from our study were only applicable to some selected patients, and it was unknown whether patients who met our exclusion criteria could benefit from early drain tube removal after LA. For example, immunocompromised patients may have an adverse impact on the surgical outcomes. In a series of cancer patients with appendicitis, many of whom were neutropenic at presentation, the majority (62.5%) were treated with antibiotics alone, 25% went straight to appendectomy (25). Another study on frail geriatric patients reported that these immunocompromised patients had more complications and higher mortality and appendectomy should be managed for a better outcome (26). Mulita also reported successful management of appendectomy for a patient with leukemia and acute appendicitis (27). Different drainage tube removal strategies in these patients may result in different surgical outcomes. Further research is needed to find out the optimal timing of drain tube removal from these excluded patients.

## Conclusions

It is safe and effective to remove abdominal drainage tube within 48 h after LA in some selected individuals with perforated appendicitis, and early removal of the drainage tube is associated with a lower rate of postoperative complications, shorter LOS and less hospitalization costs.

## Data Availability

The original contributions presented in the study are included in the article/Supplementary Material, further inquiries can be directed to the corresponding author.

## References

[1] Moris D Paulson EK Pappas TN. Diagnosis and management of acute appendicitis in adults: a review. JAMA. (2021) 326(22):2299–311. 10.1001/jama.2021.20502 34905026

[2] Gomes CA Nunes TA Fonseca Chebli JM Junior CS Gomes CC. Laparoscopy grading system of acute appendicitis: new insight for future trials. Surg Laparosc Endosc Percutan Tech. (2012) 22(5):463–6. 10.1097/SLE.0b013e318262edf1 23047394

[3] Collins CM Davenport DL Talley CL Bernard AC. Appendicitis grade, operative duration, and hospital cost. J Am Coll Surg. (2018) 226(4):578–83. 10.1016/j.jamcollsurg.2017.12.046 29391281

[4] Ukai T Shikata S Takeda H Dawes L Noguchi Y Nakayama T Evidence of surgical outcomes fluctuates over time: results from a cumulative meta-analysis of laparoscopic versus open appendectomy for acute appendicitis. BMC Gastroenterol. (2016) 16:37. 10.1186/s12876-016-0453-0 26979491 PMC4793521

[5] Di Saverio S Podda M De Simone B Ceresoli M Augustin G Gori A Diagnosis and treatment of acute appendicitis: 2020 update of the WSES Jerusalem guidelines. World J Emerg Surg. (2020) 15(1):27. 10.1186/s13017-020-00306-3 32295644 PMC7386163

[6] Neogi S Banerjee A Panda SS Ratan SK Narang R. Laparoscopic versus open appendicectomy for complicated appendicitis in children: a systematic review and meta-analysis. J Pediatr Surg. (2022) 57(3):394–405. 10.1016/j.jpedsurg.2021.07.005 34332757

[7] Kumar SS Collings AT Lamm R Haskins IN Scholz S Nepal P SAGES Guideline for the diagnosis and treatment of appendicitis. Surg Endosc. (2024) 38(6):2974–94. 10.1007/s00464-024-10813-y 38740595

[8] Walędziak M Lasek A Wysocki M Su M Bobowicz M Myśliwiec P Risk factors for serious morbidity, prolonged length of stay and hospital readmission after laparoscopic appendectomy—results from Pol-LA (Polish laparoscopic appendectomy) multicenter large cohort study. Sci Rep. (2019) 9(1):14793. 10.1038/s41598-019-51172-2 31616053 PMC6794313

[9] Liao YT Huang J Wu CT Chen PC Hsieh TT Lai F The necessity of abdominal drainage for patients with complicated appendicitis undergoing laparoscopic appendectomy: a retrospective cohort study. World J Emerg Surg. (2022) 17(1):16. 10.1186/s13017-022-00421-3 35300711 PMC8928608

[10] Neville JJ Aldeiri B. Drain placement in paediatric complicated appendicitis: a systematic review and meta-analysis. Pediatr Surg Int. (2023) 39(1):171. 10.1007/s00383-023-05457-3 37031267

[11] Alabbad J Alhamly H Alrubaiaan A Kabli A Abdulraheem F. The utility of intraperitoneal drain placement after laparoscopic appendectomy for perforated appendicitis in postoperative intraperitoneal abscess prevention. Surg Endosc. (2024) 38(7):3571–7. 10.1007/s00464-024-10869-w 38750172

[12] Wu H Liao B Cao T Ji T Luo Y Huang J Advantages comparison of peritoneal drainage versus no drainage after laparoscopic appendectomy for complicated appendicitis: a meta-analysis. BMC Gastroenterol. (2024) 24(1):411. 10.1186/s12876-024-03500-8 39550531 PMC11568599

[13] Coccolini F Improta M Sartelli M Rasa K Sawyer R Coimbra R Acute abdomen in the immunocompromised patient: WSES, SIS-E, WSIS, AAST, and GAIS guidelines. World J Emerg Surg. (2021) 16(1):40. 10.1186/s13017-021-00380-1 34372902 PMC8352154

[14] Sahara K Tsilimigras DI Moro A Mehta R Hyer JM Paredes AZ Variation in drain management among patients undergoing major hepatectomy. J Gastrointest Surg. (2021) 25(4):962–70. 10.1007/s11605-020-04610-w 32342262

[15] Dai M Liu Q Xing C Tian X Cao F Tang W Early drain removal is safe in patients with low or intermediate risk of pancreatic Fistula after pancreaticoduodenectomy: a multicenter, randomized controlled trial. Ann Surg. (2022) 275(2):e307–14. 10.1097/SLA.0000000000004992 34117153

[16] Mulita F Plachouri KM Liolis E Kehagias D Kehagias I. Comparison of intra-abdominal abscess formation after laparoscopic and open appendectomy for complicated and uncomplicated appendicitis: a retrospective study. Wideochir Inne Tech Maloinwazyjne. (2021) 16(3):560–5. 10.5114/wiitm.2021.103942 34691306 PMC8512505

[17] Mao BP Collins G Ayeni FE Vagg DJ. Risk factors for developing intra-abdominal abscess following appendicectomy for acute appendicitis: a retrospective cohort study. Langenbecks Arch Surg. (2024) 409(1):246. 10.1007/s00423-024-03421-w 39120614 PMC11315757

[18] Pakula AM Skinner R Jones A Chung R Martin M. Role of drains in laparoscopic appendectomy for complicated appendicitis at a busy county hospital. Am Surg. (2014) 80(10):1078–81. 10.1177/000313481408001036 25264664

[19] Human MJ Tshifularo N Mabitsela M. Laparoscopic appendectomy for complicated appendicitis in children: does the post-operative peritoneal drain make any difference? A pilot prospective randomised controlled trial. Pediatr Surg Int. (2022) 38(9):1291–6. 10.1007/s00383-022-05155-6 35771234 PMC9355919

[20] Fujishiro J Fujiogi M Hirahara N Terui K Okamoto T Watanabe E Abdominal drainage at appendectomy for complicated appendicitis in children: a propensity-matched comparative study. Ann Surg. (2021) 274(6):e599–604. 10.1097/SLA.0000000000003804 31977513

[21] Tang Y Liu J Bai G Cheng N Deng Y Cheng Y. Abdominal drainage to prevent intraperitoneal abscess after appendectomy for complicated appendicitis. Cochrane Database Syst Rev. (2025) 4(4):CD010168. 10.1002/14651858.CD010168.pub5 40214287 PMC11987584

[22] Li Z Li Z Zhao L Cheng Y Cheng N Deng Y. Abdominal drainage to prevent intra-peritoneal abscess after appendectomy for complicated appendicitis. Cochrane Database Syst Rev. (2021) 8(8):CD010168. 10.1002/14651858.CD010168.pub4 34402522 PMC8407456

[23] Liao J Zhou J Wang J Xie G Wei H. Prophylactic abdominal drainage following appendectomy for complicated appendicitis: a meta-analysis. Front Surg. (2023) 9:1086877. 10.3389/fsurg.2022.1086877 36743896 PMC9889918

[24] Voglino V Frediani S Aloi IP Pardi V Bertocchini A Accinni A Use of drainage after laparoscopic complicated appendectomy in children: a single-center experience. Minerva Pediatr (Torino). (2024). 10.23736/S2724-5276.24.07483-4 39297421

[25] Santos D Chiang YJ Badgwell B. Appendicitis in cancer patients is often observed and can represent appendiceal malignancy. Am Surg. (2016) 82(10):1028–32. 10.1177/000313481608201038 27779999

[26] Chehab M Ditillo M Khurrum M Gries L Asmar S Douglas M Managing acute uncomplicated appendicitis in frail geriatric patients: a second hit may be too much. J Trauma Acute Care Surg. (2021) 90(3):501–6. 10.1097/TA.0000000000003028 33617197

[27] Mulita F Oikonomou N Provatidis A Alexopoulos A Maroulis I. Roseomonas gilardii in patient with leukemia and acute appendicitis: case report and review. Pan Afr Med J. (2020) 36:283. 10.11604/pamj.2020.36.283.24834 33117477 PMC7572676

